# Sealing the Pores of PEO Coating with Mg-Al Layered Double Hydroxide: Enhanced Corrosion Resistance, Cytocompatibility and Drug Delivery Ability

**DOI:** 10.1038/s41598-017-08238-w

**Published:** 2017-08-15

**Authors:** Feng Peng, Donghui Wang, Yaxin Tian, Huiliang Cao, Yuqin Qiao, Xuanyong Liu

**Affiliations:** 1 State Key Laboratory of High Performance Ceramics and Superfine Microstructure, Shanghai Institute of Ceramics, Chinese Academy of Sciences, Shanghai, 200050 China; 2 University of Chinese Academy of Sciences, Beijing, 100049 China

## Abstract

In recent years, magnesium (Mg) alloys show a promising application in clinic as degradable biomaterials. Nevertheless, the poor corrosion resistance of Mg alloys is the main obstacle to their clinical application. Here we successfully seal the pores of plasma electrolytic oxidation (PEO) coating on AZ31 with Mg-Al layered double hydroxide (LDH) via hydrothermal treatment. PEO/LDH composite coating possess a two layer structure, an inner layer made up of PEO coating (~5 μm) and an outer layer of Mg-Al LDH (~2 μm). Electrochemical and hydrogen evolution tests suggest preferable corrosion resistance of the PEO/LDH coating. Cytotoxicity, cell adhesion, live/dead staining and proliferation data of rat bone marrow stem cells (rBMSCs) demonstrate that PEO/LDH coating remarkably enhance the cytocompatibility of the substrate, indicating a potential application in orthopedic surgeries. In addition, hemolysis rate (HR) test shows that the HR value of PEO/LDH coating is 1.10 ± 0.47%, fulfilling the request of clinical application. More importantly, the structure of Mg-Al LDH on the top of PEO coating shows excellent drug delivery ability.

## Introduction

With favorable properties of biodegradation and mechanical behaviors, Mg alloys are an attractive choice for orthopedic applications^1–3^. However, along with the fast degradation of Mg, an increasing pH value will be observed in the microenvironment and cause the inflammation of tissues^4, 5^. More seriously, the loss of mechanical strength might result in the failure of implantation. Surface modification is one of the most effective methods to enhance the corrosion resistance of Mg alloys, including hydrothermal treatment^6–8^, plasma electrolytic oxidation (PEO)^9–14^, electron beam treatments^15^, ion implantation^16^, apatite coating^11^, and organic polymer coating^12, 17, 18^ etc. PEO as one of the most commonly studied methods can produce a highly adherent ceramic oxide coating, endowing Mg alloys an obviously improved corrosion resistance. However, pores on the surface formed during the PEO process limited its corrosion resistance, because corrosive solution could easily penetrate into the substrate through the pores. Cui *et al*. reported that coating thickness has an insignificant influence on the corrosion resistance of PEO coating, while porosity of the coating is one of the main factors that determine its corrosion resistance^19^.

Many studies have been focused on decreasing number and size of pores on the surface of PEO coating to improve its corrosion resistance^20–22^. Zhao *et al*. added graphene oxide into the electrolyte and found that the number of pores on the PEO coating was significantly decreased^22^. Moreover, some researchers tried to fabricate self-sealing PEO coating on Mg alloys^9, 10, 13^. Dong *et al*. used electrolyte consisted of NaH_2_P_2_O_7_, K_2_TiF_6_, NaF, C_6_H_12_N_4_ and NaOH, to prepare self-sealing PEO coating on AM60^10^. However, there were still few pores and cracks on such PEO coating. As long as PEO coating contains pores or cracks, its long-term corrosion resistance cannot be guaranteed. Considering these situations, researchers fabricate composite coatings to seal the pores of PEO coating. Li *et al*. used hydroxyapatite to seal the pores of PEO coating and found that osseointegration and corrosion resistance of Mg alloy are enhanced^23^. Our previous study also revealed that poly(L-lactide) can be used to sealed the pores of PEO coating and enhanced its corrosion resistance and biocompatibility^12^. However, both hydroxyapatite and poly(L-lactide) are unable to work as a drug delivery platform.

Layered double hydroxides (LDHs), with a chemical formula [M^2+^_1−x_M^3+^x(OH)_2_][A^n−^]_x/n_·zH2O, where M^2+^ represent bivalent cations, M^3+^ represent trivalent cations, are made up of positively charged brucite-like layers and an interlayer region containing various anions and solvation molecules. The special structure of LDHs makes it a desirable platform for drug delivey^24–27^. Li *et al*. employed LDH nanoparticles to simultaneously deliver an anticancer drug 5-fluorouracil (5-FU) and Allstars Cell Death siRNA (CD-siRNA) to overcome the drug resistance and enhance cancer treatment^28^. Furthermore, our previous study revealed that Mg alloy coated with Mg-Al LDH exhibit desirable biocompatibility^29^. On the other hand, the small particle size of LDH exactly can be applied to seal the pores of PEO coating. From these perspectives, we explore the way to use Mg-Al LDH to seal the pores of PEO coating to enhance its corrosion resistance. Moreover, LDH, as the outer layer, could improve the biocompatibility of Mg alloy and endow its surface a drug-delivery ability to regulate cells’ behaviors.

**Figure 1:**
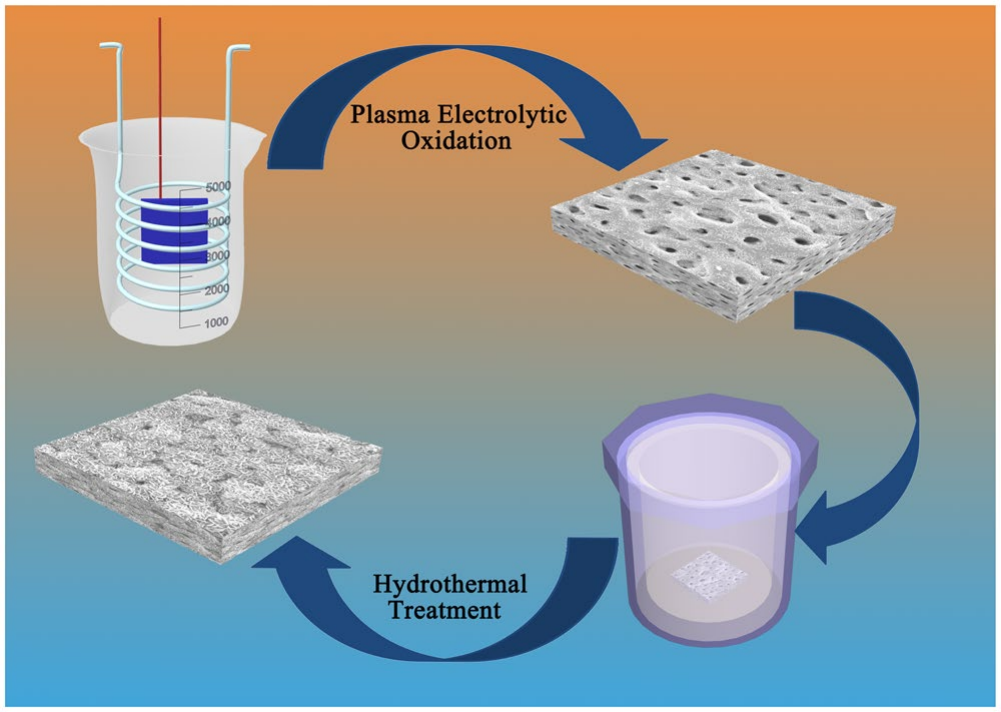
The process of fabricating PEO/LDH coating.

Herein, we *in-situ* grew Mg-Al LDH on PEO coating to seal its pores. As shown in Fig. 1, PEO coating incorporated with fluoride was firstly produced via a PEO process, and followed by a hydrothermal treatment. As PEO coating would release Mg ions to the solution, Mg-Al LDH can be *in-situ* formed on the top of PEO coating. Thus a newly designed PEO/LDH composite coating was acquired. Corrosion resistance, cytocompatibility, hemolysis rate and drug loading ability of PEO/LDH composite coating were evaluated subsequently.

## Results and Discussions

### Coating Characterization

**Figure 2:**
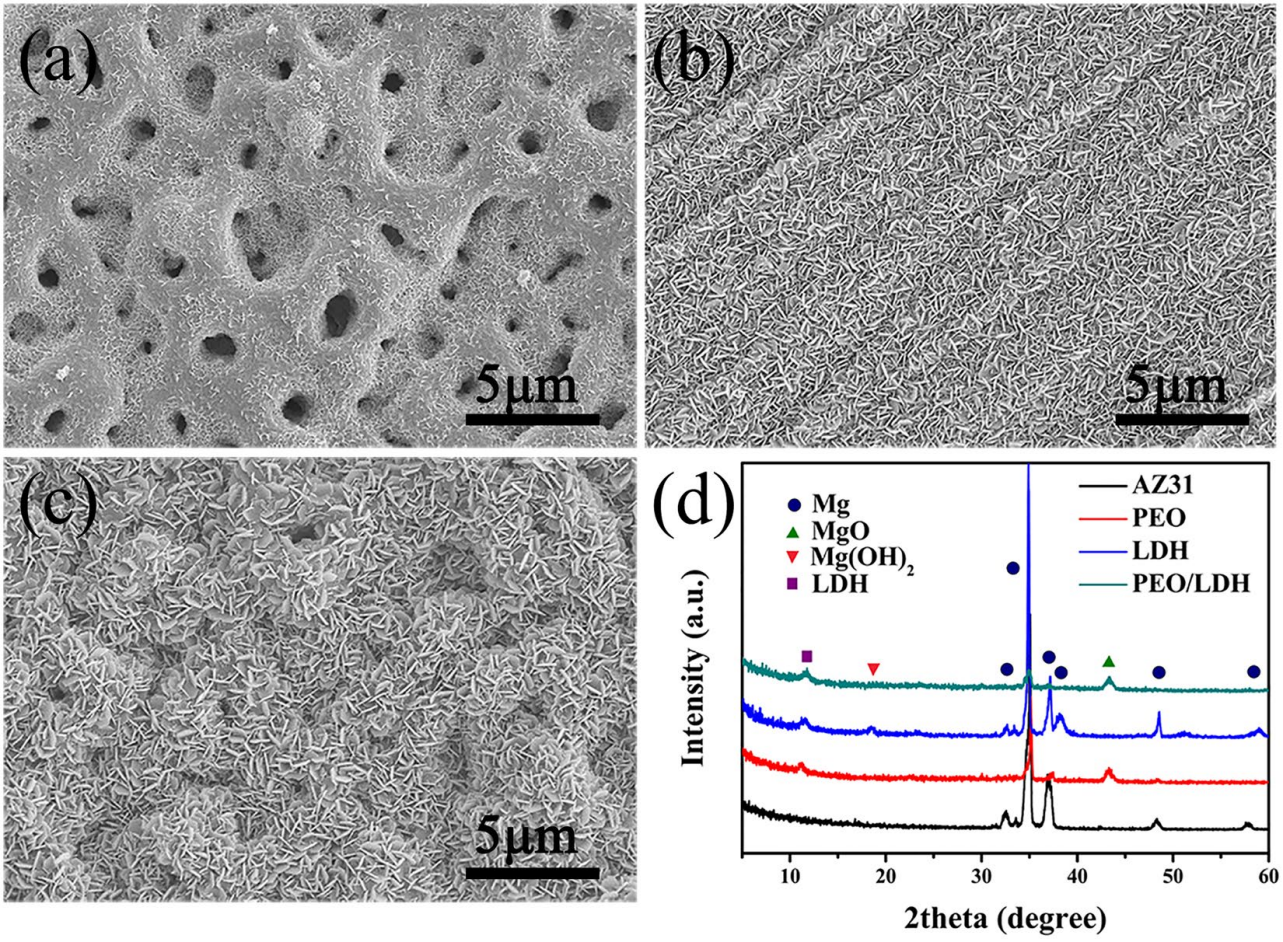
Surface morphology of PEO (**a**), LDH (**b**) and PEO/LDH (**c**); XRD patterns of all samples (**d**).

Figure 2a–c depict the surface morphology of three coated samples. PEO coating showed porous structure and a trace of nano-sheets closely adhered to its surface (Fig. 2a). A compacted nanoflake-like structure appeared on the surface of LDH and PEO/LDH coating (Fig. 2b,c). With regard to PEO/LDH coating, the homogeneous and compacted nanoflake-like structure was formed on the top of PEO coating after hydrothermal treatment, successfully sealing the pores of PEO coating. Furthermore, both nanoflake-like structure of LDH and PEO/LDH coating showed superior attachment to the substrate (Figure S1 in the Supporting Information). The XRD patterns of all samples are shown in Fig. 2d. Only feature peaks of Mg were detected in the pattern of AZ31 alloy. In the pattern of PEO coating, the crystalline phase of MgO appeared and the diffraction peak around 11.7° indicated the formation of Mg-Al LDH. The result certifies that the nano-sheet observed on the surface of PEO coating is Mg-Al LDH. As AZ31 contains Mg and Al element, Mg^2+^ and Al^3+^ ions would release from the substrate during the PEO process, then reacted with OH^−^ and formed Mg-Al LDH. Both Mg(OH)_2_ and Mg-Al LDH were detected in the pattern of LDH, which is consistent with our previous study^29^. It is worth mentioning that there was no Mg(OH)_2_ phase observed on the surface of PEO/LDH coating, which means the nanoflake-like structure on its surfaces was pure Mg-Al LDH. The Mg-Al LDH peaks appeared in the patterns of PEO, LDH and PEO/LDH were around 11.7°, corresponding to (003) crystal plane, and revealing an interlayer spacing of 0.76 nm.

**Figure 3:**
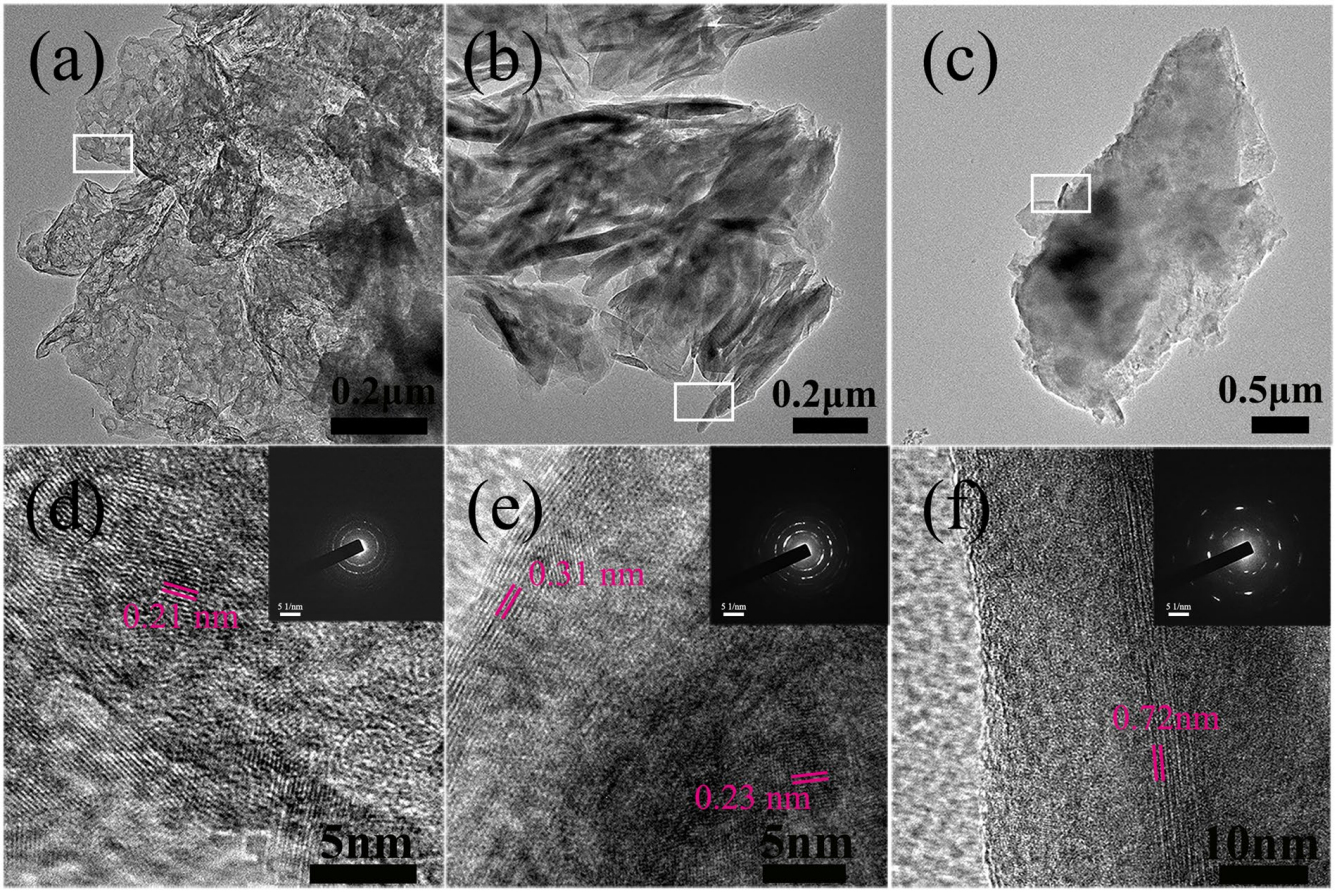
TEM analysis results of morphology, HRTEM and SAED of PEO (**a**,**d**), LDH (**b**,**e**) and PEO/LDH (**c**,**f**).

To conduct TEM analysis, powder was scratched off from the surface of specimen, and the result is also displayed in Fig. 3. Figure 3a shows typical bright-field TEM images of PEO coating powder. The corresponding high-resolution image in Fig. 3d clearly revealed a set of fringes in different directions and the measured interplanar spacing was 0.21 nm, representing (200) lattice plane of MgO. The high-resolution image (Fig. 3e) of LDH powder suggested two set of fringes. The fringe with 0.31 nm interplanar spacing was ascribed to (006) lattice plane of Mg-Al LDH and the fringe of 0.23 nm to (101) lattice plane of Mg(OH)_2_. With regard to PEO/LDH specimen powder, as shown in Fig. 3f, fringe with 0.72 nm interplanar spacing indicated (003) lattice plane of Mg-Al LDH. The polycrystalline nature of all the powders was demonstrated by the continuous rings in the selected area electron diffraction (SAED) pattern in the inset of Fig. 3d–f. These results are consistent with the XRD patterns.

**Figure 4:**
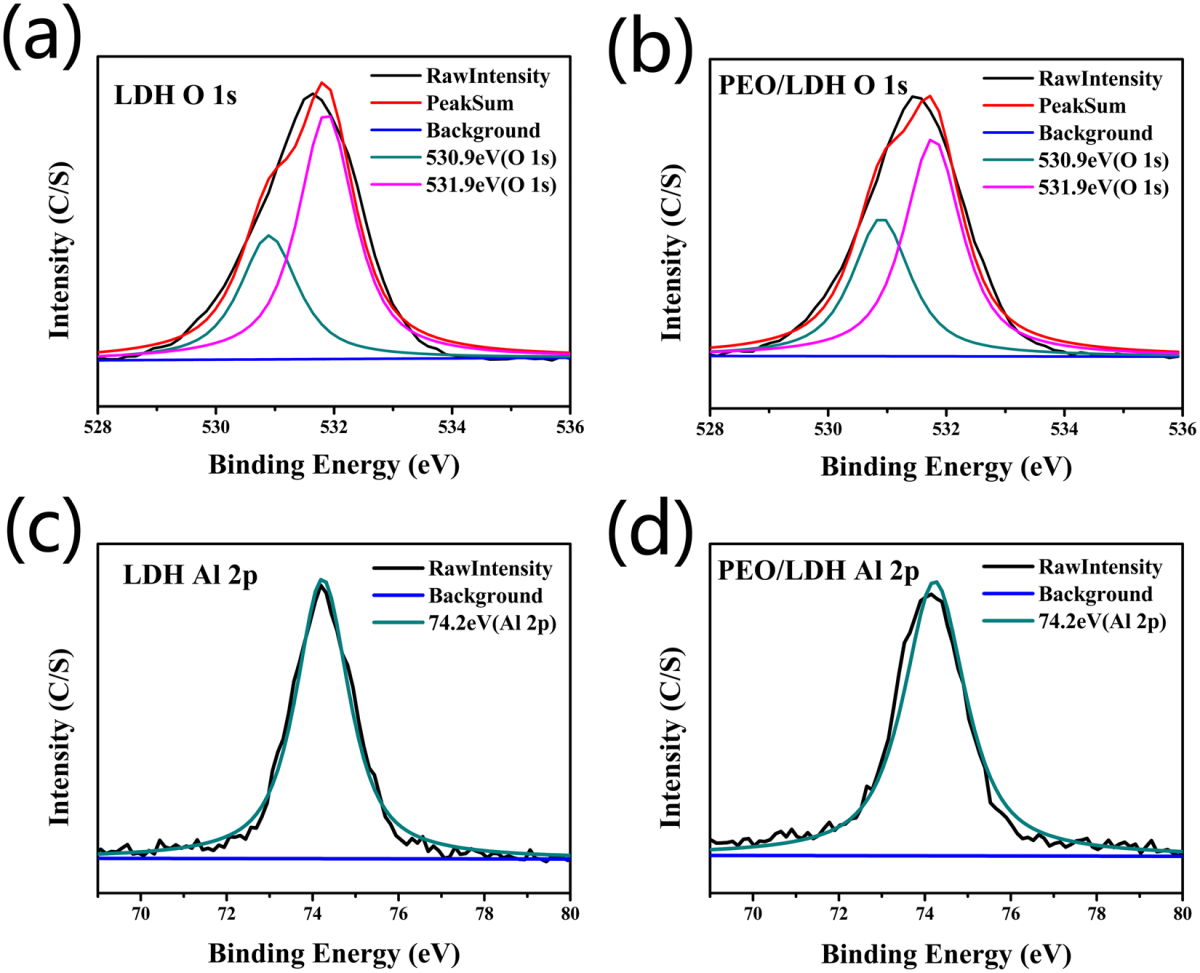
High-resolution spectra of O 1 s of LDH (**a**) and PEO/LDH (**c**), Al 2p of LDH (**b**) and PEO/LDH (**d**).

Element composition and chemical states of surface elements were carried out using XPS. Table 1 shows the surface element composition of all coatings. Al element was detected on all samples. However, the content of Al on PEO coating (0.41%) was obviously lower than the other two samples (5.44% and 5.57%, respectively), indicating that only few Mg-Al LDH were formed on the surface of PEO during the PEO process as shown in Fig. 2a. N element was detected on the surface of LDH and PEO/LDH coatings, suggesting that nitrate was in the interlayer of Mg-Al LDH to balance the positive charge. High-resolution spectra of O 1 s and Al 2p of LDH and PEO/LDH are show in Fig. 4a–d. Both of the O 1 s peak can be divided into two peaks centered at 530.9 and 531.9 eV corresponding to oxygen peak in hydroxyl bonding with Mg and Al. Al 2p peak of LDH and PEO/LDH centered at 74.2 eV ascribed to aluminum peak bonding with hydroxyl. The results of XPS further confirmed the existence of Mg-Al LDH on all treated samples.

**Table 1:** Surface elemental composition of coated samples measured by XPS.

Samples	C (at %)	N (at %)	O (at %)	Mg (at %)	Al (at %)	Al/Mg
PEO	11.89	/	59.48	28.22	0.41	0.015
LDH	22.83	0.36	56.67	14.7	5.44	0.37
PEO/LDH	30.02	0.43	49.86	15.1	4.57	0.302

**Figure 5:**
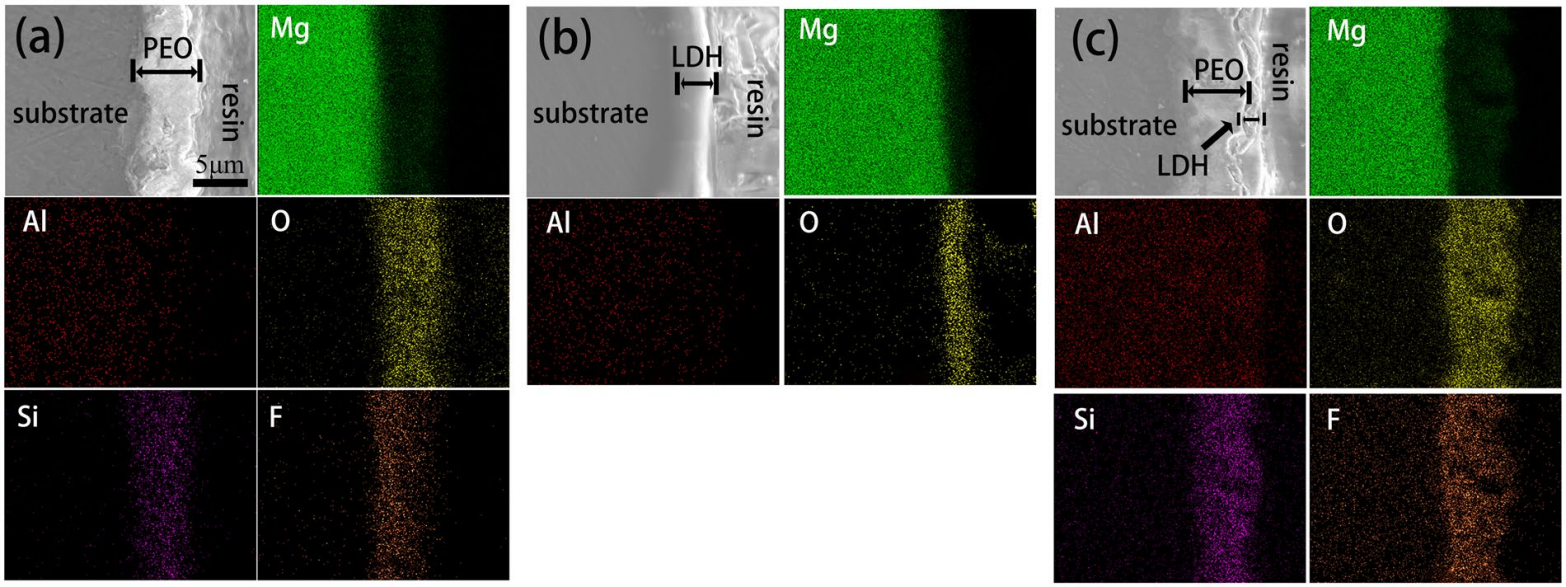
Cross-sectional morphology of PEO (**a**), LDH (**b**) and PEO/LDH (**c**), and corresponding scanning maps of characteristic elements.

The cross-sectional morphology of treated samples and corresponding scanning maps are presented in Fig. 5a–c. The thickness of PEO and LDH coating were approximately 6.5 μm and 3.5 μm, respectively. There were two layers of PEO/LDH coating. The inner layer ascribed to the process of PEO, while the outer layer ascribed to hydrothermal treatment, and the thickness were about 5 μm and 2 μm, respectively. During the process of hydrothermal treatment, part of the PEO coating gradually dissolved in the alkaline hydrothermal solution, releasing Mg^2+^ ions^30, 31^. The released Mg^2+^ reacted with OH^−^ and AlO_2_^−^, forming Mg-Al LDH particles in the pores and on the surface of PEO coating. As the reaction proceeding, Mg-Al LDH finally sealed the pores of the PEO coating. Thus, a two layer structure coating was fabricated. In addition, the content of fluorine on the surface of PEO/LDH was also decreased comparing to PEO coating (Table S1). The corresponding scanning maps of Mg, Al, O, Si and F (Fig. 5c) further confirmed the double layer structure of PEO/LDH coating.

### Corrosion Resistance

**Figure 6:**
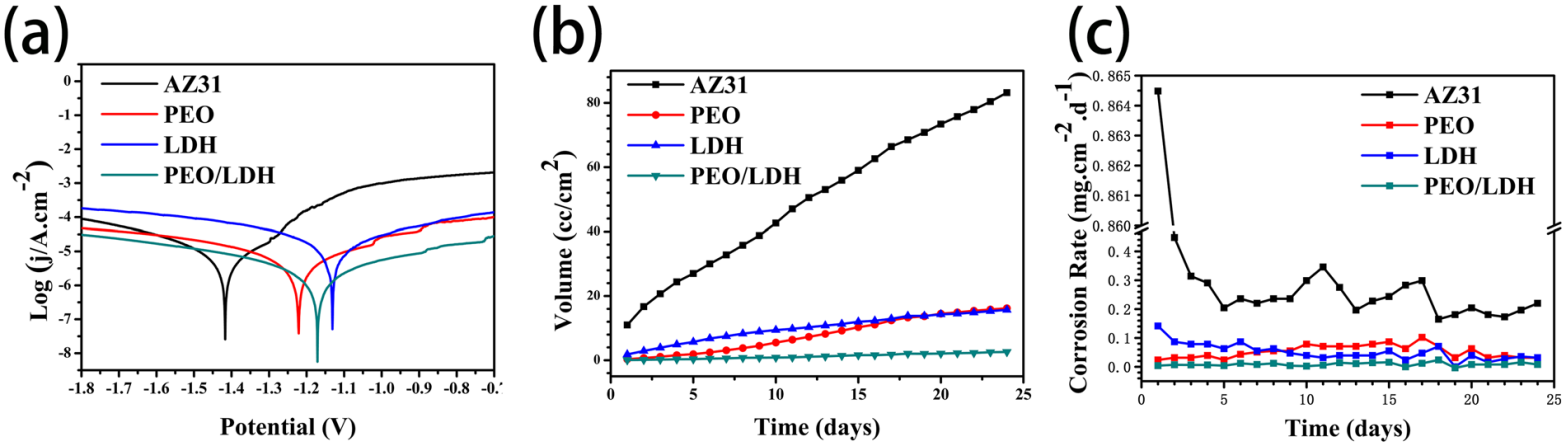
Polarization curves (**a**), hydrogen evolution (**b**) and corrosion rate (**c**) of all samples.

Figure 6a describes the electrochemical potentiodynamic polarization curves of all samples and corresponding corrosion parameters are showed in Table 2. Comparing to AZ31 and PEO, LDH and PEO/LDH showed lower free current density j_corr_ and higher corrosion potential E_corr_. Though the corrosion potential E_corr_ of PEO/LDH was slightly lower than that of LDH, the polarization resistance R_p_ of PEO/LDH was about one order higher than LDH, implying an enhanced protective effect. The result suggests that PEO/LDH composite coating possesses the best corrosion resistance.

**Table 2:** Corrosion potential (E_corr_), corrosion current density (i_corr_) and polarization resistance (Rp) calculated according to the polarization curves.

Samples	AZ31	PEO	LDH	PEO/HT
j_corr_ (A·cm^−2^)	1.66 × 10^−5^	0.945 × 10^−5^	3.34 × 10^−5^	3.92 × 10^−6^
E_corr_ (V)	−1.45	−1.22	−1.12	−1.2
R_p_ (Ω·cm^−2^)	7.21 × 10^4^	1.13 × 10^5^	3.05 × 10^4^	2.79 × 10^5^

According to the corrosion reaction of Mg + H_2_O → Mg^2+^  + OH^−^ + H_2_ (gas), both the hydrogen evolution and the change of pH value can reflect the corrosion rate of the sample^32, 33^. Figure 6b and c exhibit the hydrogen evolution and the corrosion rate of all samples. The quick hydrogen releasing of AZ31 alloy was obvious suppressed after surface modification. PEO showed a slower degradation rate than LDH at the start of corrosion. After corroding for 9 d, the corrosion rate of PEO exceeded LDH. PEO/LDH showed the least hydrogen evolution and lowest corrosion rate, indicating that Mg-Al LDH on the top of PEO coating can effectively protect the substrate from corroding. The result of pH value changes (Figure S2) is in agreement with that of hydrogen evolution test. The above results suggest that PEO/LDH coating manifested a most protective ability for the substrate. Many studies ascribed the collapse of PEO coating mainly to the pores on its surface^34^. Thus, it is vital for the coating on the surface of Mg to be compacted and without pores, especially at the initial corrosion stage. *In-situ* growing a compacted Mg-Al LDH layer on the top of the PEO coating could seal the pores, preventing corrosive medium penetrate into the substrate through the pores and resulting in a more effective protection for the substrate.

### Biological Performances

**Figure 7:**
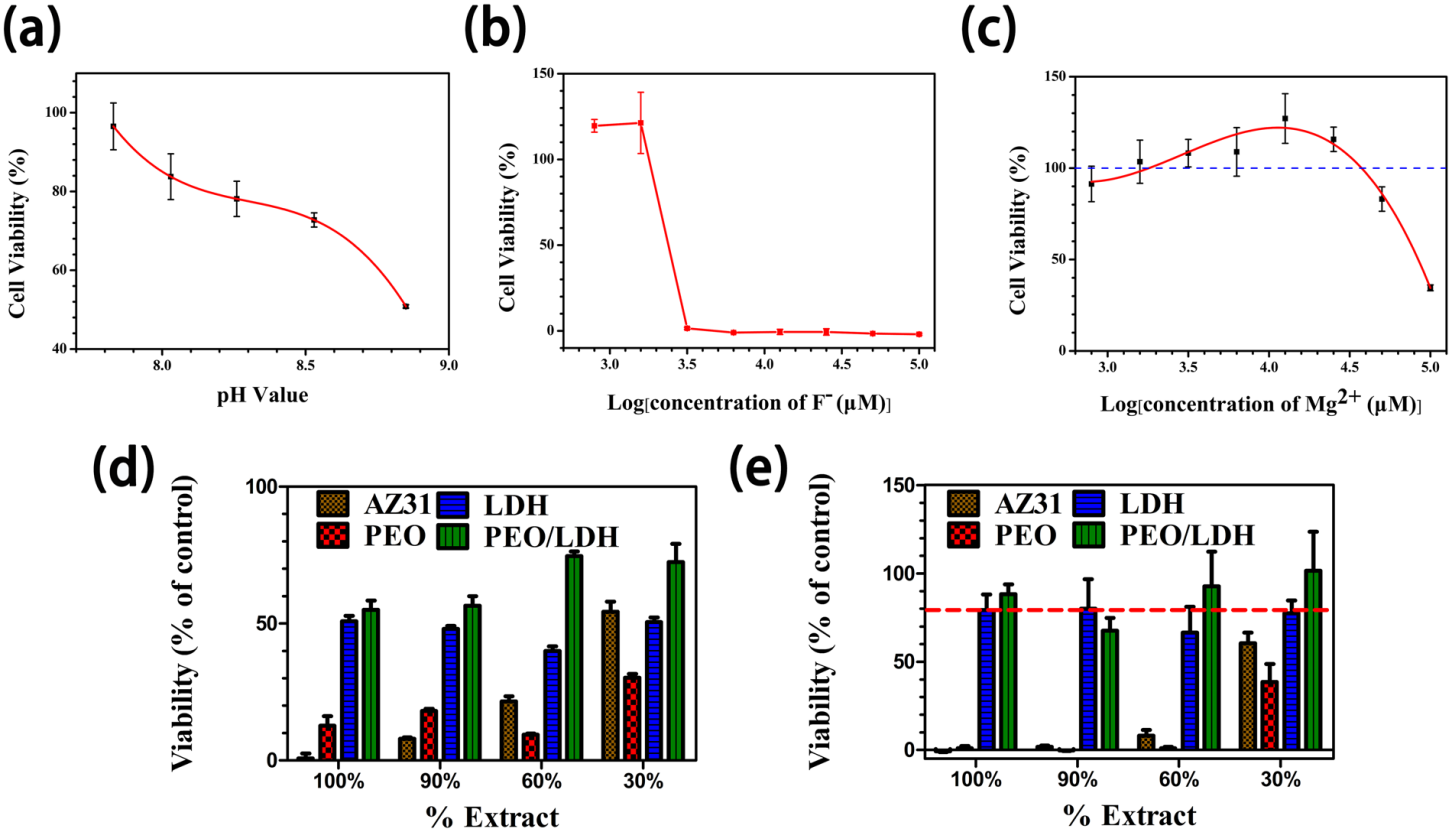
The influence of pH values (**a**), concentration of F ions (**b**) and Mg ions (**c**) to cell viability. Viability of cells incubated for 1 d (**d**) and 4 d (**e**) with different concentration extract of all samples.

The degradation of sample would lead to a serious change of culture medium, such as excessive Mg and F ions, increased pH value. Bearing this situation in mind, we investigate the influence of these factors to cell viability independently, and the results are showed in Fig. 7.

The pH value of human tissue is in the range of 7~7.4. A high pH value beyond this optimal range would influence cellular metabolism^35^. Here, in the range of pH 7.6~8.8, the cell viability was found to be inversely proportional to the pH value (Fig. 7a). Cell displayed a high sensitivity to F ions (Fig. 7b), with the fact that the cell viability sharply decreased to zero when the concentration of F ions reached 3.125 mM. The result conforms to our previous study that PEO coating with a high content of fluoride would cause great damage to cells^36^. As Mg ions concentration increased, the cell ability first increasing and then decreasing (Fig. 7c). On the one hand, Mg ions can take part in various physiological intracellular reactions^37^. Therefore, a moderate concentration of Mg ions will be favorable for cell proliferation. On the other hand, Mg ions are closely related to the osmolality of the culture medium. As a consequence, excessive Mg ions in the medium will significantly elevate the culture medium osmolality^38^, and the drawback of extortionate osmolality will override the positive impact of Mg ions on the cell viability.

Figure 7d and e exhibit the viability of cell cultured in different extract for 1 and 4 d, respectively. After incubating for 1 d, all the extracts showed severe cytotoxicity (<60%), especially the extract of AZ31 (2%) and PEO (15%). However, when incubation time extended to 4 d, the viability of cells in LDH and PEO/LDH extract was larger than 80%, suggesting slight cytotoxicity. In contrast, cells in the extract of AZ31 and PEO were totally died. Table 3 shows the pH value, concentration of Mg and F ions of the extract. Both pH value and Mg ions concentration of AZ31 extract were highest among all the extract, explaining the highest cytotoxicity of AZ31 extract. As for PEO, the pH value and Mg ions concentration decreased while F ions showed up. The F ions concentration were up to 4.74 mM, beyond the threshold value (3.125 mM), and resulted in a huge damage to cells. Applying hydrothermal treatment on PEO coating would reduce the F content of the coating (Table S1), and the formation of Mg-Al LDH would inhibit the release of F ions. Thus, the F concentration of PEO/LDH extract (2.42 mM) was only half of the PEO extract and under the threshold value, supporting its slight cytotoxicity (with a cell viability of 90% after 4 d). The cell viability in extract of LDH which contained excessive Mg ions was slightly inferior to that of PEO/LDH, mainly owing to its significant change of osmolality caused by poorer corrosion resistance.

**Table 3:** The pH value, concentration of F and Mg ions of the extracts and culture medium.

	Culture medium	AZ31	PEO	LDH	PEO/LDH
pH	7.6	8.43	8.09	8.03	8.05
F^−^ (mM)	—	—	4.74	—	2.42
Mg^2+^ (mM)	0.81	10.53	9.29	9.24	4.95

**Figure 8:**
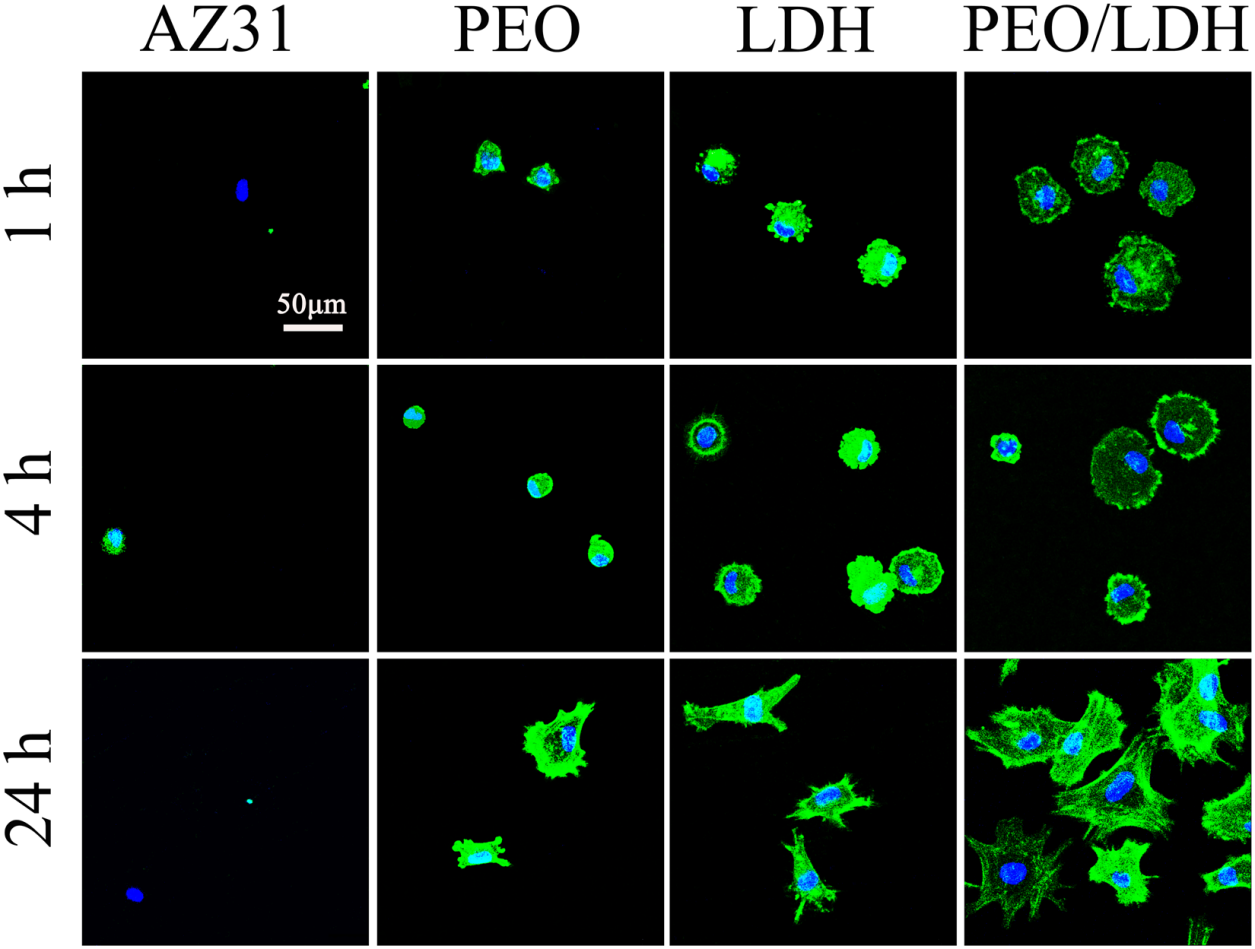
CLSM images of cells cultured on various surfaces for 1, 4 and 24 h with actin stained with FITC (green) and nucleus stained with DAPI (blue).

The initial cell adherence process was observed by CLSM and the result is displayed in Fig. 8. At the first hour, hardly any cell adhered on the surface of AZ31, while a certain number of cells adhered onto other samples and showed a round morphology, especially for PEO/LDH, on which cells displayed a more spreading morphology. No obvious changes were observed after 4 h incubation. However, after 24 h, cell morphology on coated samples transferred to polygon and cells on PEO/LDH showed a more spread morphology with numerous filopodia and lamellipodia than those on PEO and LDH. In addition, a larger number of cells were observed on the surface of PEO/LDH than other samples.

The results suggest that PEO/LDH coating could enhance the cell adhesion and spreading, presenting a positive affinity to cells. A further confirmation was provided by MC3T3-E1 cells which also showed an enhanced adhesion on the surface of PEO/LDH (Figure S3).

**Figure 9:**
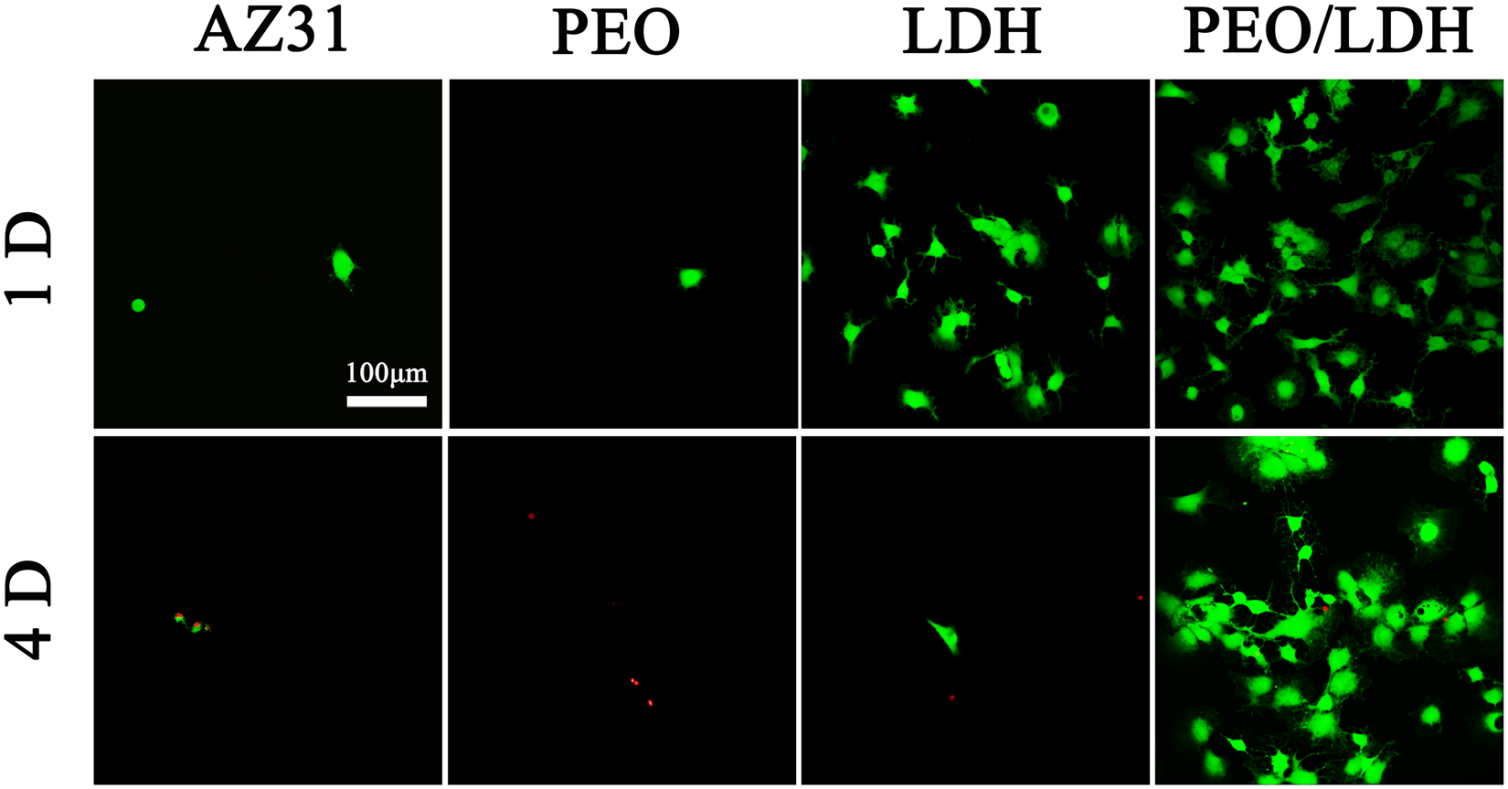
CLSM images of live/dead staining of cell after culturing on various surfaces for 1 and 4 d (green represent live cells and red represent dead cells).

Figure 9 shows the results of live/dead staining. Nearly no living cells were detected on the surfaces of AZ31 and PEO after 1 d. However, a large number of living cells were observed on the surface of LDH and PEO/LDH, especially on PEO/LDH. After culturing for 4 d, cells on LDH were almost died, while cells on PEO/LDH still remain alive. Furthermore, cells on PEO/LDH could maintain their viability even after 14 d (Figure S4). To further verify the potential application of the prepared films on orthopedic surgeries, we also evaluate the viability of MC3T3-E1 cells cultured on different samples. The results suggested that MC3T3-E1 cells could survive on LDH and PEO/LDH (Figure S5).

The fact that both rBMSCs and MC3T3-E1 cells can successfully adhere and proliferate on the surface of PEO/LDH sample implies a promising application of PEO/LDH composite coating in orthopedic surgeries.

**Figure 10:**
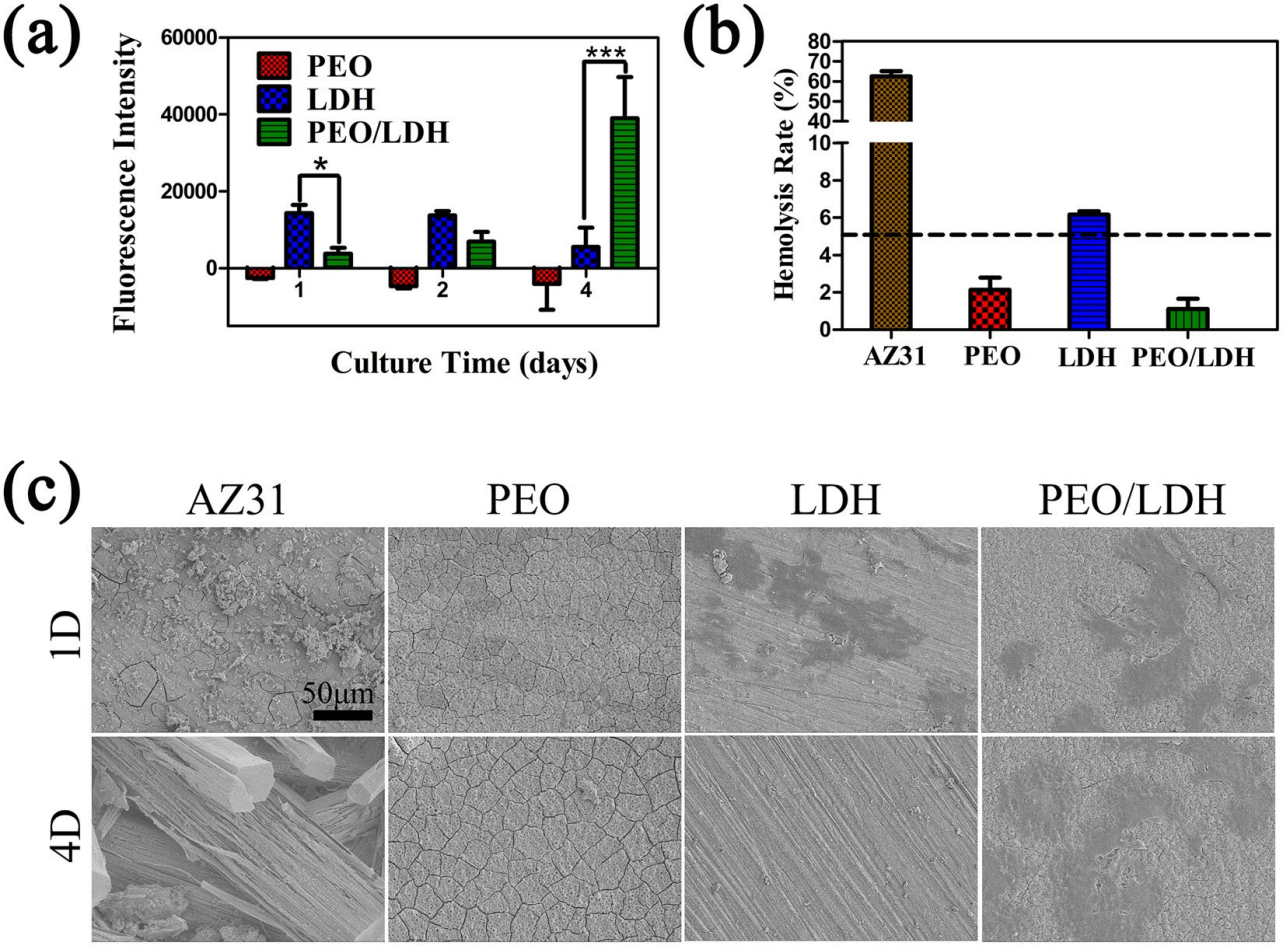
The proliferation rate (**a**) and morphology (**c**) of cells cultured on various surfaces; Hemolysis rate of all samples (**b**).

The quantitative result of cell proliferation cultured on coated samples is depicted in Fig. 10a and corresponding cell morphology in Fig. 10c. Cell proliferation on AZ31 wasn’t measured, given that (i) the results of cell adhesion and live/dead staining indicate that there are no living cells on the surface of AZ31 after culturing for 4 d, (ii) a large number of released Mg ions from the substrate and pH change of the culture medium would lead to a false positive result^38^. Over the incubation period, there were no cells observed on PEO coating because of plethora of F ions released from the sample. Cells on LDH showed the highest proliferation in the first 2 d. However, cell number on PEO/LDH enormously increased at 4 d, significantly exceeding that on LDH. At initial culture stage, F ions released from PEO/LDH was suppressed in comparison to PEO, but still possibly do harm to cells, resulting in a lower proliferation rate compared with LDH. After 4 d, the influence of F ions to cells on PEO/LDH alleviated, and the excessive Mg ions released from LDH would inhibit the viability of cells. Cell morphology (Fig. 10c) clearly showed that cells could only survive on the surface of PEO/LDH after culturing for 4 d, demonstrating PEO/LDH is more suitable for cell growth and proliferation. The result further confirms the promising application of PEO/LDH composite coating in orthopedic surgeries.

Hemolysis rate (HR), reflecting the degree of erythrocyte destruction, is a pivotal characterization for blood-contacting materials. As shown in Fig. 10b, HR values of PEO, LDH and PEO/LDH were 2.13 ± 0.71%, 6.16 ± 0.16%, 1.10 ± 0.47%, respectively, which were substantially lower than AZ31 (62.35 ± 3%). The HR value of the sample is closely related to its corrosion behavior and a better corrosion resistance result in a lower HR value^39^. As showed in the result of corrosion rate (Fig. 6c), the corrosion resistance of the samples (PEO/LDH å PEO å LDH å AZ31) exactly in agreement with the HR value. Furthermore, the HR values of PEO and PEO/LDH were low enough for clinical application (5% is an acceptable value for clinical application).

### Drug Delivery

**Figure 11:**
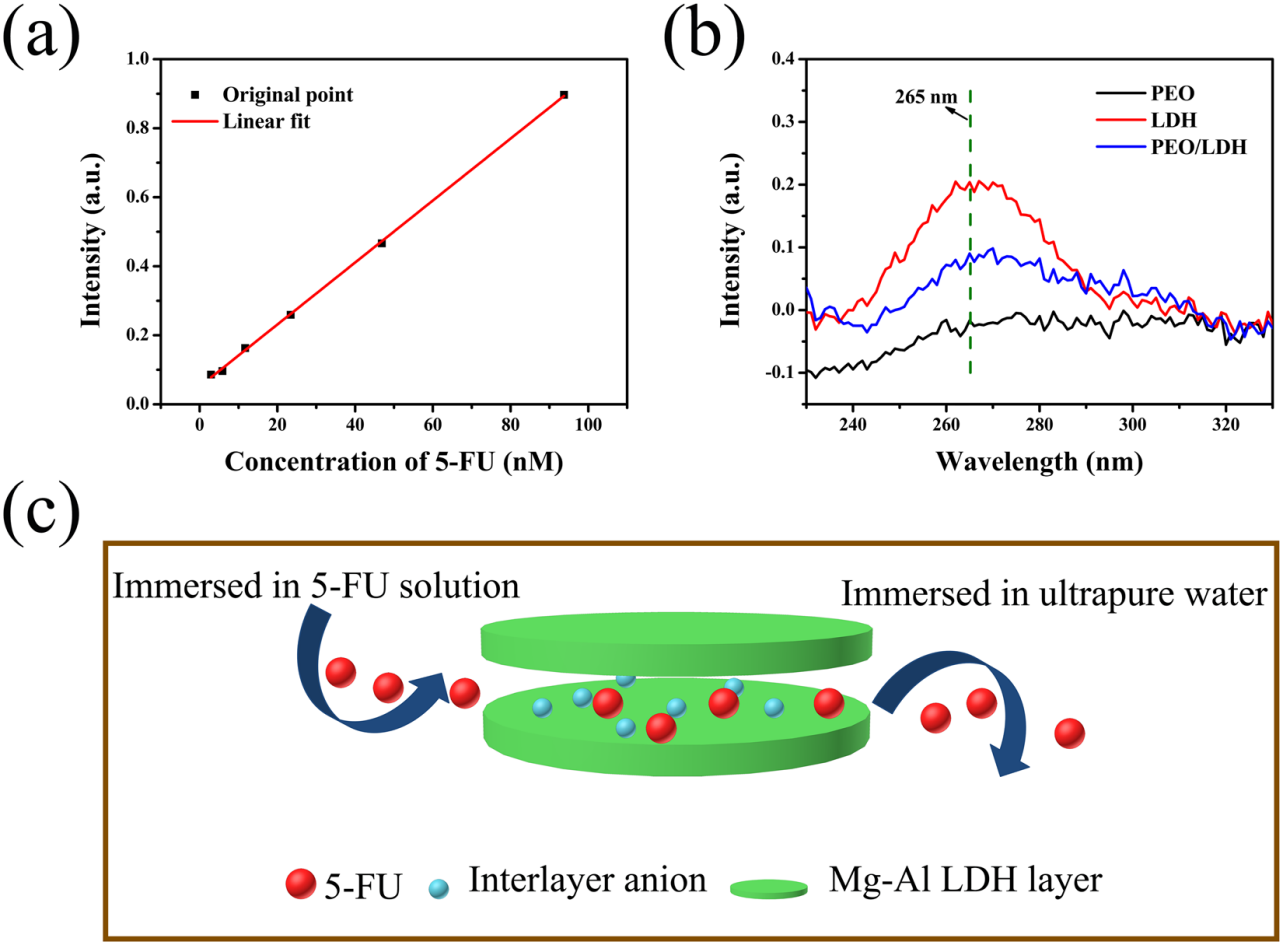
The standard curve of 5-FU (**a**), the ultraviolet absorption spectrum of the solution after releasing 5-FU, and the process of loading-releasing 5-FU (**c**).

LDHs-based drug delivery systems have been studied for many years^24, 28^. However, almost all these LDHs are in the form of powder. In this work, we fabricated Mg-Al LDH layer on PEO coating and its drug loading ability was measured. The standard curve of 5-FU is showed in Fig. 11a. The concentration of 5-FU between 0–100 nM is proportional to its intensity of ultraviolet (UV) absorption at 265 nm. After loading 5-FU, samples were immersed in ultrapure water, and the UV absorption spectrum of the resulting solution is showed in Fig. 11b. There was no 5-FU released from PEO. The amount of 5-FU released from LDH (20.58 ± 0.73 nM) was almost three times of PEO/LDH (7.46 ± 0.25 nM). According to the atomic ratios of Al (Table 1) and the thickness of the coatings (Fig. 5), the amount of Mg-Al LDH structure on LDH is more than that on PEO/LDH, explaining the greater drug loading ability of LDH. Figure 11c depicts the process of loading-releasing 5-FU. When immersed in 5-FU solution, the interlayer anions (mainly consist of hydroxyl, nitrate and carbonate) of Mg-Al LDH would be replaced by 5-Fu via an ion-exchange process. The loaded 5-FU would release into the surrounding when immersed in an environment without 5-FU. The efficiency of the newly designed drug release platform will be investigated in our future work.

## Conclusions

In summary, we have successfully developed a PEO/LDH composite coating on the surface of AZ31. The pores of PEO coating were sealed via *in-situ* growing Mg-Al LDH. The corrosion resistance of the substrate was remarkably enhanced by PEO/LDH coating. Moreover, the adhesion and proliferation of rBMSCs were improved. The HR value of PEO/LDH coating was decreased to a clinical application. Finally, the Mg-Al LDH endowed PEO/LDH composite coating a favorable drug delivery ability. The prepared composite coating in this study shows a promising application in orthopedic surgeries.

## Materials and Methods

### Coating Fabrication

#### Preparation of PEO Coating

The PEO film was grown on AZ31 as previous reported^36^. Briefly, commercial AZ31 was cut into 10 mm × 10 mm × 2 mm and ground with 1000# SiC abrasive paper, and then ultrasonically cleaned with ethyl alcohol, dried in the air. The process was conducted by PEO equipment (Pulsetech, China) with the constant current density of 0.3 A/cm^2^, frequency of 800 Hz and duty cycle of 10%. The process was stopped at a voltage of 360 V. The electrolyte contained 0.04 M Na_2_SiO_3_·9H_2_O, 0.1 M KOH and 0.2 M KF·2H_2_O (samples obtained were denoted as PEO).

#### Preparation of LDH Coating

AZ31 alloy samples and PEO samples were placed in a Teflon-lined stainless at 120 °C for 12 h. The reaction solution were 0.02 M aluminum nitrate (pH = 12.8, adjusted by NaOH). After hydrothermal treatment, obtained samples were denoted as LDH and PEO/LDH, respectively.

### Coatings Characterization

The surface and cross-sectional morphologies of all the coatings were observed by scanning electron microscopy (SEM; Hitachi-S3400N, Hitachi, Japan), and elemental compositions of the samples surfaces were evaluated by energy dispersive spectrometry (EDS; IXRF-550i, IXRF SYSTEMS, USA). Meanwhile, scanning maps of Mg, O, Al, Si, F were also measured by EDS. The phase compositions of AZ31 alloy, PEO, LDH and PEO/LDH were analyzed by X-ray diffraction (XRD; D/Max, RIGAKU, Tokyo, Japan). TEM analysis was performed using a field emission transmission electron microscope (TEM; JEM-2100F, JEOL Ltd, Tokyo, Japan). The chemical states of surface elements were measured by X-ray photoelectron spectroscopy (XPS, PHI-5000C ESCA system PerkinElmer, USA).

### Corrosion Behavior Evaluation

#### Electrochemical test

Electrochemical corrosion of all samples was tested using a CHI760C electrochemical analyzer (Shanghai, China) in phosphate buffer saline (PBS). The process was conducted in a three-electrode electrochemical cell with a saturated calomel electrode (SCE) as the reference electrode, a graphite rod as the counter electrode. Ahead of the test, samples were stabilized in PBS for 400 seconds. The test was conducted at a scanning rate of 10 mV/s with a temperature of 37 °C. The corrosion potential (*E*_corr_), current density (*i*_corr_) and polarization resistance (R_p_) were calculated according to Tafel extrapolation.

#### Hydrogen evolution test

For hydrogen evolution test, six parallel samples for each group were placed in 360 mL PBS at 37 °C. The volume of released hydrogen was recorded up to 24 days. According to the volume of hydrogen, the corrosion rate of tested samples can be calculated via the following formula:

1 $$r=\frac{PV}{RT}\times \frac{M}{A{\rm{t}}}$$

where r is the corrosion rate (mg·cm^−2^·d^−1^), P is standard atmospheric pressure (Pa), V is volume of H_2_ (mL), R is 8.314 J/(mol·K), T is the temperature (K), M is the molar mass (g/mol), A is the original surface area (cm^2^), t is the exposure time (day).

### Cytocompatibility

The rat bone marrow stem cells (rBMSCs) were used to evaluate the cytocompatibility of all samples. Cells mentioned in this work refer to rBMSCs if not specified. Cells were cultured with α-MEM (Minimum Essential Medium alpha-Medium) at 37 °C in a humidified atmosphere of 5% CO_2_ in air.

#### Cytotoxicity evaluation

After sterilized by ultraviolet irradiation, samples were incubated in α-MEM for 24 h with volume/surface ratio of 0.5 cm^2^/mL. The extracted solution was designated as 100%, diluted to 90%, 60% and 30% with α-MEM. Meanwhile, 100 μL cell suspensions with a cell density of 5 × 10^4^ cell/mL were added to each well of a 96-well culture plate. After 24 h, 100 μL extracted solution with different concentration replace the culture medium and incubated for another 1 and 4 d. α-MEM without extract served as the control group. Cells viability was tested by the alamarBlue assay (AbD Serotec Ltd, UK) according to the manufacturer’s instruction, and calculated using the following equation:

2 $${\rm{Viability}}=\frac{{F}_{S}}{{F}_{C}}\times 100 \% $$

where F_s_ is the fluorescence intensity of the sample group and F_c_ is the fluorescence intensity of the control group.

#### Cell adhesion

Cells were seeded on samples at a density of 3 × 10^4^ cells/well. After 1, 4 and 24 h, samples were rinsed with PBS. Then cells were fixed, permeabilized and blocked successively by 4% paraformaldehyde (PFA) diluent, 0.1% (v/v) Triton X-100 (Amresco, USA) and 1 wt % bovine serum protein (BSA, Sigma, USA) respectively. Then FITC phalloidin was added to stain F-actin, and DAPI stain nucleus. Samples were rinsed with PBS after each step. Finally, specimens were observed by confocal laser scanning microscopy (CLSM, Leica SP8, Germany).

#### Live/Dead Cell Staining

The live/dead cell staining kit (Biovision, USA) was used according to manufacturer’s instructions. Briefly, cells were seeded on the samples with a density of 3 × 10^4^ cells/well, cultured for 1 and 4 d. Then propidium iodide (PI) and calcium-AM were diluted to final concentrations of 5 and 2 μM in PBS, respectively. 100 μL of mixed solution was added to each sample, and cells were observed by CLSM after stained at 37 °C for 15 min.

#### Cell proliferation and morphology

Cells were seeded on samples with a density of 3 × 10^4^ cells/mL. The proliferation rates of the cells were determined by alamarBlue assay at 1, 2 and 4 d. After 1 and 4 d incubation, samples cultured with cells were dehydrated in a grade ethanol series (30, 50, 75, 90, 95, and 100% v/v) for 10 min, respectively, with final dehydration conducted in absolute ethanol twice, followed by drying in the hexamethyldisilizane ethanol solution series (1: 2, 2: 1, pure hexamethyldisilizane). Cells morphology was detected by SEM.

### Hemolysis Rate Test

The human blood was obtained from healthy adult donors. Samples were placed in a 24-well plate with 1.5 mL 0.9 wt% NaCl solution and kept at 37 °C for 30 min. Untreated 0.9 wt% NaCl and distilled water served as negative and positive controls respectively. After that, the solution was replaced with 30 μL diluted blood (0.8 mL whole blood was diluted with 1 mL 0.9 wt% NaCl solution) and incubated for 1 h. Subsequently, the solution was centrifuged at 3000 rpm for 5 min. The optical density of the supernatant was measured at 545 nm by an enzyme-labeling instrument. The HR was calculated using the following equation:

3 $$HR=\frac{A{S}_{545}-A{N}_{545}}{A{P}_{545}-A{N}_{545}}\times 100 \% $$

where AS_545_ is the absorption value of the samples, AN_545_ is the absorption value of the negative control and AP_545_ is the absorption value of the positive control.

### Drug Delivery Evaluation

5-Fluorouracil (5-FU, Sigma, USA) was used in this test. To transport 5-FU into the interlayer of Mg-Al LDH, samples (PEO, LDH and PEO/LDH) were placed in a 24-well plate, and immersed in 1 mL 5-FU solution (6 μM) for 4 h at 37 °C. After that, samples were rinsed with plenty of deionized water, and dry in ambient. Subsequently, samples were immersed in 1 mL ultrapure water to release 5-FU. The amount of released 5-FU was determined according to the standard curve, measured by UV-VIS-NIR Spectrometer (PerkinElmer, USA).

## Electronic supplementary material

Supplementary Information
