# Supplementary material for: Sealing the Pores of PEO Coating with Mg-Al Layered Double Hydroxide: Enhanced Corrosion Resistance, Cytocompatibility and Drug Delivery Ability
Source: Sci Rep. 2017 Aug 15;7:8167. doi: 10.1038/s41598-017-08238-w (PMC5557750; doi:10.1038/s41598-017-08238-w)
Supplement: Supplementary file 1 — Supplementary Information [file 41598_2017_8238_MOESM1_ESM.doc]

**Supporting Information**

**Sealing the Pores of PEO Coating with Mg-Al Layered Double Hydroxide: Enhanced Corrosion Resistance, Cytocompatibility and Drug Delivery Ability**

Feng Peng^1,2^, Donghui Wang^1,2^, Yaxin Tian^1,2^, Huiliang Cao^1^, Yuqin Qiao^1^, Xuanyong Liu^1^,*

^1^ State Key Laboratory of High Performance Ceramics and Superfine Microstructure, Shanghai Institute of Ceramics, Chinese Academy of Sciences, Shanghai 200050,China.

^2^ University of Chinese Academy of Sciences, Beijing 100049, China.

**^*^Corresponding Author**

Prof. Xuanyong Liu.

E-mail: [xyliu@mail.sic.ac.cn](mailto:xyliu@mail.sic.ac.cn).

Tel.: +86 2152412409. Fax: +86 21 52412409.


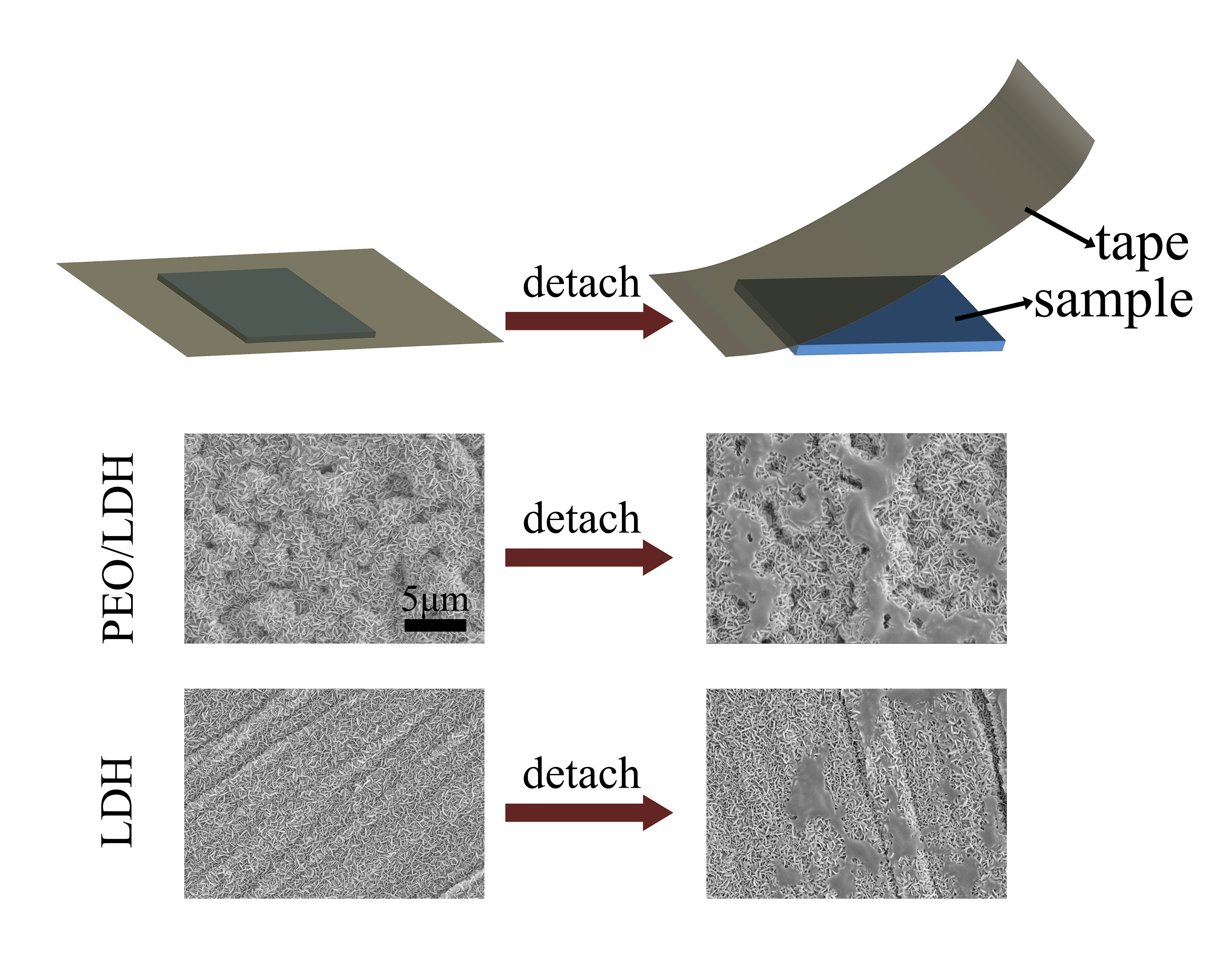


**Figure S1.** Surface morphology of the samples before and after the tape detached.

**Table S1.** Surface compositions of different samples measured by EDS.

|  | O (wt %) | | F (wt %) | Mg (wt %) | Al (wt %) | Si (wt %) | Zn (wt %) |
| --- | --- | --- | --- | --- | --- | --- | --- |
| AZ31 | | 3.42 | / | 92.16 | 3.54 | / | 0.88 |
| PEO | | 41.90 | 12.11 | 36.38 | 1.34 | 7.46 | 0.76 |
| LDH | | 61.01 | / | 34.83 | 3.62 | / | 0.54 |
| PEO/LDH | | 51.67 | 4.69 | 31.52 | 5.65 | 5.7585 | 0.72 |


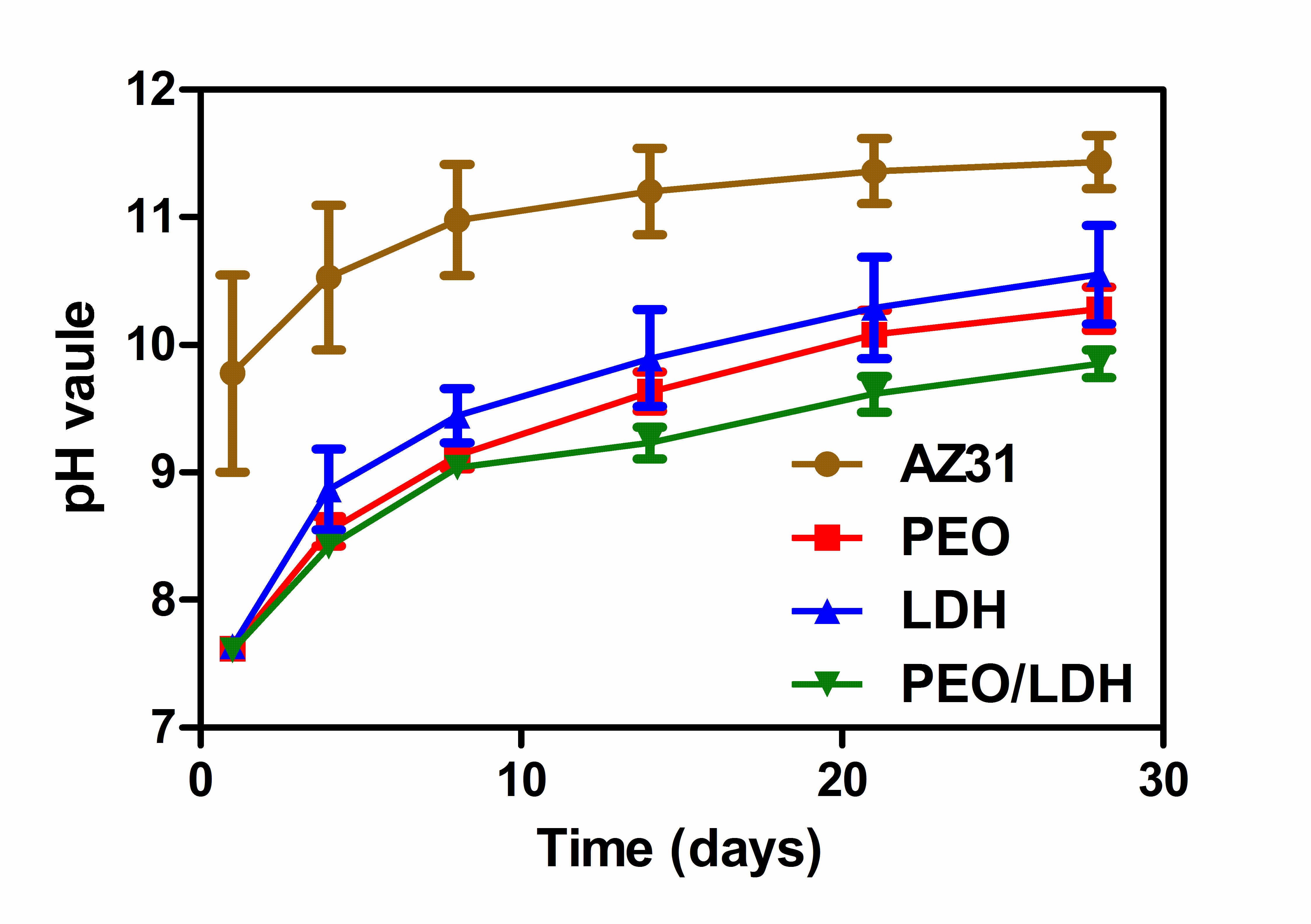
 **Figue S2.** pH value changes of all samples.


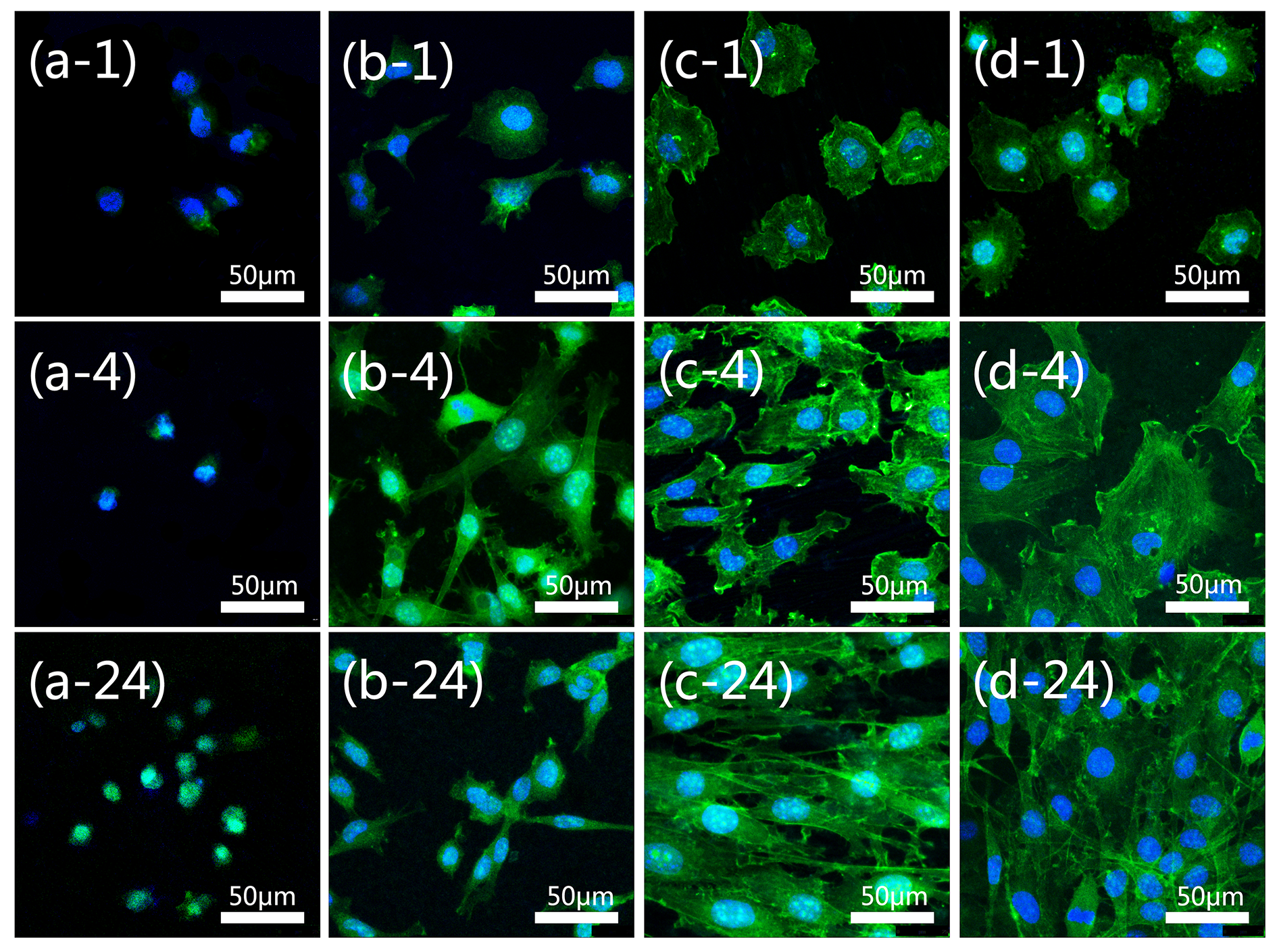


**Figure S3.** CLSM images of MC3T3-E1 cells cultured on AZ31 alloy (a), PEO (b), LDH (c) and PEO/LDH (d) for 1 h (i-1), 4 h (i-4) and 24 h (i-24) (i stands for a, b, c and d) with actin stained with FITC (green) and the nucleus stained with DAPI (blue).


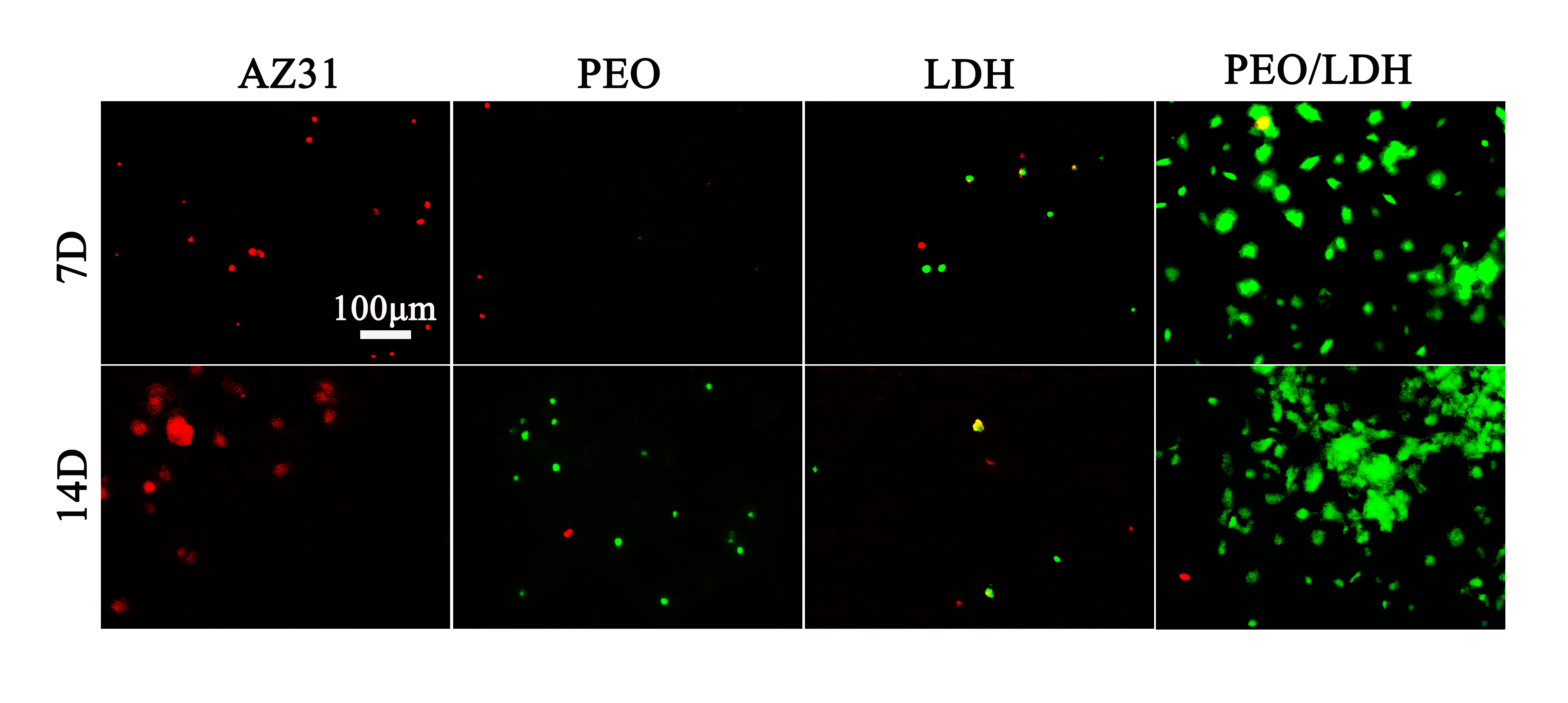


**Figure S4.** CLSM images of live/dead staining of rBMSCs after culturing on various surfaces for 7 and 14 d (green represent live cells and red represent dead cells).


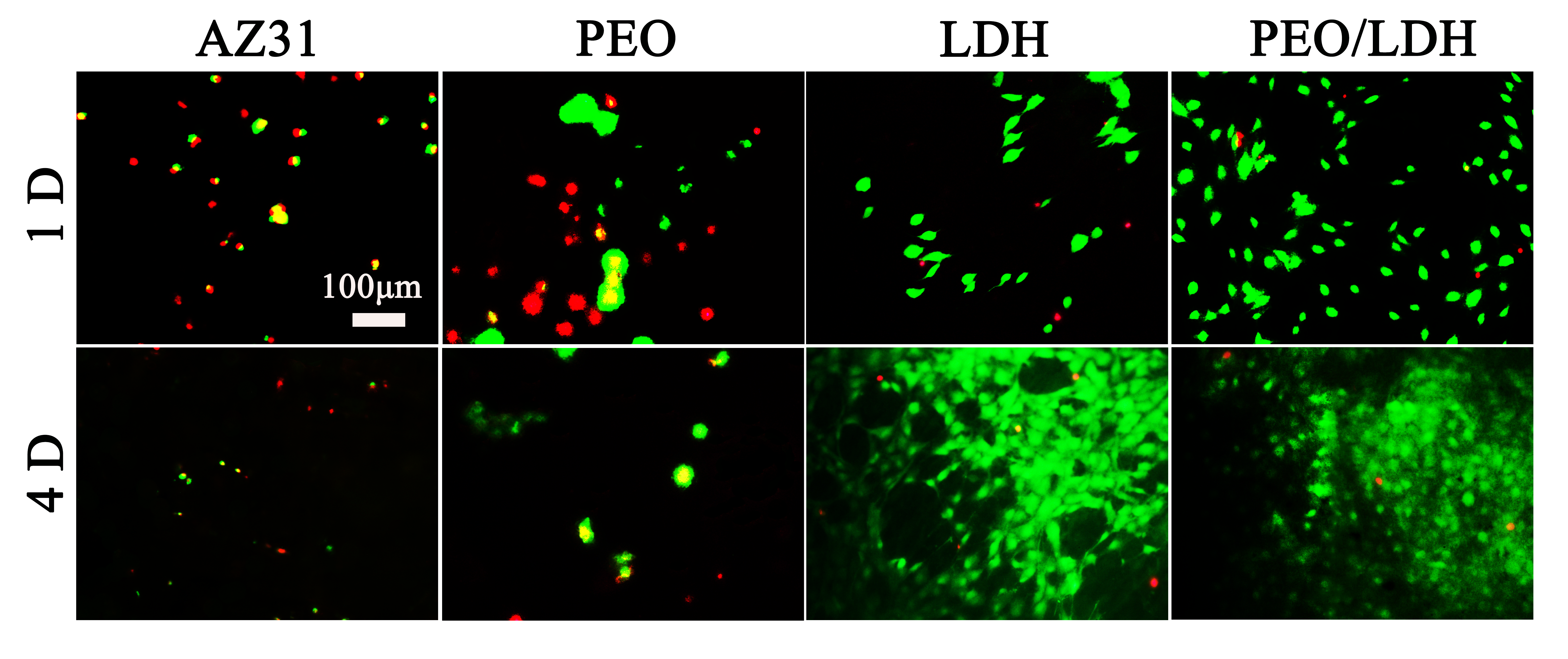


**Figure S5.** CLSM images of live/dead staining of MC3T3-E1 cells after culturing on various surface for 1 and 4 d (green represent live cells and red represent dead cells).
